# The Influence of Dietary Chicken Egg Lysozyme on the Growth Performance, Blood Health, and Resistance Against *Escherichia coli* in the Growing Rabbits' Cecum

**DOI:** 10.3389/fvets.2020.579576

**Published:** 2020-10-15

**Authors:** Mahmoud H. EL-Deep, Khairy A. Amber, Yahya Z. Eid, Sara T. Alrashood, Haseeb A. Khan, Mohamed S. Sakr, Mahmoud A. O. Dawood

**Affiliations:** ^1^Animal Production Research Institute, Agricultural, Research Center, Ministry of Agriculture, Giza, Egypt; ^2^Department of Poultry Production, Faculty of Agriculture, Kafrelsheikh University, Kafr El-Sheikh, Egypt; ^3^Department of Pharmaceutical Chemistry, College of Pharmacy, King Saud University, Riyadh, Saudi Arabia; ^4^Department of Biochemistry, College of Science, King Saud University, Riyadh, Saudi Arabia; ^5^Department of Animal Production, Faculty of Agriculture, Kafrelsheikh University, Kafr El-Sheikh, Egypt

**Keywords:** lysozyme additives, growing rabbits, blood constituents, caecal microbiota, hematology

## Abstract

The dietary chicken egg lysozyme (LZM) at different concentrations was tested on the growth performance, blood health, and resistance against Escherichia coli of growing rabbits. A total number of 48 rabbits averaged 611.25 g (5 weeks of age) of APRI line-rabbits (Egyptian developed line) were allocated into four treatments (three replicates and each contained four rabbits) of 5-week weaning APRI rabbits. The first group was fed a basal diet without LZM supplementation and served as a control group, whereas the remaining groups of rabbits were fed a basal diet supplemented with LZM at 50, 100, and 200 mg/kg diet, respectively, for 8 weeks. The obtained results revealed that rabbits fed the basal diet supplemented with different concentrations of LZM linearly (*P* < 0.05) displayed improved growth performance and reduced feed intake and FCR. The best result was for rabbits fed a 200 mg per kg diet supplemented with LZM, followed by a 100 mg per kg diet. The total count of *Escherichia coli* and *Clostridium* count was linearly (*P* < 0.05) decreased by adding LZM at 100 and 200 mg/kg in the diets compared to the control groups. In contrast, total bacterial count and the total count of *Lactobacilli* had increased considerably by increasing LZM at different levels relative to the control groups. The LZM supplementation linearly (*P* < 0.05) increased hematological parameters (RBCs, PCV, Hb, and WBCs) together with an increase in lymphocyte count compared to the control group. The total protein and globulin concentrations were significantly (*P* < 0.05) increased by feeding with LZM. On the other hand, ALT, AST, urea, and creatinine were significantly (*P* < 0.05) decreased by increasing LZM supplementation. It could be concluded that supplementation of the rabbit's diet with chicken egg LZM was able to improve the growth performance and hematological and serum biochemical parameters compared with the control group. Therefore, LZM is required at the rate of the hobx100-200 mg/kg diet as a potential feed additive and a friendly alternative for antibiotics in rabbit feed.

## Introduction

Rabbit meat offers excellent nutritive and dietetic properties for the consumers' needs as a rich source for animal protein (20–21%) (1, 2). Furthermore, it has high levels of zinc, iron, selenium, potassium, magnesium, and vitamin B, as well as low cholesterol and sodium levels (3). Pursuing intensive production systems with simultaneous attendance of one or multifactorial stressors (environmental, nutritional, and managerial), this caused several negative impacts on the health status and the ability of rabbits to resist infectious diseases. In such cases, antibiotics usually are used to relieve the effects of environmental stressors and contagious diseases on rabbits (4). It is worth noting that using antibiotics and chemicals over a long time has resulted in the emergence of pathogens that became resistant to such treatment (5, 6). Thus, antibiotics have been banned in several countries around the globe because of its harmful effects on human health (7).

The continuous application of chemotherapies weakens the natural immunity of rabbits, and its prolonged usage induces accumulated amounts in animals' meat which can transfer to the human body and threatens the natural resistance against invaders (2, 8). Especially after the COVID-19 pandemic, it becomes crucial to look for “alternative friendly additives” which can replace the routine usage of drugs, antibiotics, and vaccines in the field of rabbits' production (9). Several alternatives to antibiotics have been used, such as probiotics, prebiotics, organic acids, enzymes, essential oils, and immunostimulants (5, 10–14). One possible candidate is using lysozyme (LZM), which can be used as a growth promoter in feed ingredient as a potential replacement for dietary antibiotics (15, 16). In this concern, LZM is derived from eggs, with its ability to cleave the N-acetylmuramic acid and N-acetylglucosamine detritus in Gram-positive bacteria together with its ability to restrict Gram-negative bacteria (17, 18). It presents several properties associated with antimicrobial, antitumor, antiviral, anti-histaminic, antioxidant, anti-inflammatory, and immune-modulatory peculiarities (19). Dietary LZM activated the immune response and maintained gut barrier function, promoted small intestinal morphology, modulated intestinal microbes, and attenuated stress during *Escherichia coli* K88 oral contestation (20, 21). Chicken egg LZM is widely distributed in various biological fluids and tissues, including avian egg and animal secretions; human milk, tears, saliva, and airway secretions; and those secreted by polymorphonuclear leukocytes (22).

Based on the studies mentioned above and related information, it can be hypothesized that LZM would have beneficial effects on the performances of growing rabbits. This study also exclusively investigated the impacts of LZM on growing rabbits and presented a natural alternative growth promoter to replace antibiotics. Hence, the study aimed to evaluate the effects of LZM on the growth performance, blood constituents, and cecal microbiota of growing rabbits.

## Materials and Methods

### Rabbits and Experimental Design

The experimental protocol used in this study was approved by the Animal Care and Use Committee of Kafrelsheikh University (2018-06). The trial was carried out at Sakha Poultry Research Station and Laboratories, Kafrelsheikh, Animal Production Research Institute, Egypt, during the period from September to November. A total number of 48 multicolored APRI-Line weaning male rabbits, with 611.25 g BW, 5 weeks of age, were raised under normal managerial and hygienic conditions throughout the experimental period from 5 to 13 weeks of age (8 weeks). Rabbits were raised in wire galvanized battery cages with dimensions (60 × 40 × 35 cm) and supplied with feeders and automatic stainless-steel nipples. Rabbits were equally divided into four homogeneous groups of three replicates (each cage contained four rabbits) with available space of 0.6 m^2^/head. The lighting program submitted 14 h of light per day. Averages of perimeter temperature (°C) and relative humidity were 18–20°C and 55–65%. Freshwater was available all the time through automatic nipple drinks in each cage. Feed was exhibited *ad libitum* during the trial period.

Extraction lysozyme from hen egg white (egg non-fertility) was accomplished, according to Ibrahim et al. (23). The composition of the basal diet is illustrated in Table 1, according to NRC (24). Rabbits were distributed into a completely randomized design where, in the control group, rabbits were fed without LZM supplementation, while LZM was included at 50, 100, and 200 mg per kg diet in the remaining groups, successively. The respective test diets were administered for 8 weeks. The feed intake and live body weight were recorded twice weekly, then body weight gain and feed conversion ratio were calculated.

**Table 1:** Composition and chemical analysis (dry matter basis) of the basal diet.

**Ingredients**	**%**	**Analysis**	**%**
Berseem hay	30	Dry matter	89.7
Barley	23	Crude protein	17.2
Soybean meal	17.5	Crude fiber	13.1
Wheat bran	15	Ether extract	3.4
Yellow corn	10	Nitrogen free extract	56.
Molasses	3	Ash	10.3
Di. Ca. phosphate	0.8	Digestible energy (Kcal/kg)	2,519
Sodium chloride	0.3		
Vit. & min. premix^*^	0.3		
DL-Methionine	0.1		

* *Vit. & min. The mixture provides per kilogram contains Vit. A 6,000 IU, Vit. D_3_ 450 IU, Vit. E 40 mg, Vit. K_3_ 1 mg, Vit. B_1_ 1 mg, Vit. B_2_ 3 mg, Vit. B_3_ 180 mg, Vit. B_6_ 39 mg, Vit. B_12_ 2.5 mg, pantothenic acid 10 mg, biotin 10 mg, folic acid 2.5 mg, choline chloride 1,200, manganese 15 mg, zinc 35 mg, iron 38 mg, copper 5 mg, selenium 0.1 mg, and iodine 0.2 mg*.

### Microbial Enumerations

Samples from the cecum were chosen individually from 2 rabbits per replicate (six rabbits from each group), and cecum contents were obtained after slaughtering and filtrated to estimate pH and cecum microflora. Total bacterial count, *E. coli, Clostridium*, and *Lactobacillus* count were done according to Collins and Lyne (25).

### Blood Sampling, Hematological, and Biochemistry Examination

The blood was collected from the lateral ear vein of slaughtered rabbits (2 rabbits per replicate = 6 rabbits each group) and immediately divided into two aliquots, where EDTA (ethylenediaminetetraacetic acid) was used as an anticoagulant (1 mg/ml) to collect the first aliquot for hematological parameters. The second aliquot was left to clot at room temperature and then centrifuged at 3,000 rpm for 15 min. Sera were then separated and stored at −20°C into aliquots for individual biochemical estimations.

Red blood cell (RBC) count, hemoglobin (Hb), packed cell volume (PCV), mean corpuscular volume (MCV), mean corpuscular hemoglobin concentration (MCHC), white blood cells (WBCs), and differential leukocytic count were evaluated according to Schalm et al. (26).

The serum was used to detect serum, liver, renal, and injury biomarkers [alanine aminotransferase (ALT), aspartate aminotransferase (AST), total proteins, albumin, creatinine, urea, triglycerides, and HDL-cholesterol], which were detected according to the manufacturer's protocol using commercial kits (Diamond Diagnostic, Dokki, Giza, Egypt). Globulins concentration in serum was computed by subtracting the albumin concentration from total proteins, while the albumin to globulin ratio (A/G) was calculated according to Kaneko et al. (27). LDL-C and VLDL-C concentrations were analyzed using the standard Friedewald equation (28). Glucose was analyzed according to Trinder (29) using a spectrophotometer and commercial test kits of Randox (Antrim, UK), following the manufacturer's instructions. The LDL (mg/dL) was calculated using the equation LDL = total cholesterol—HDL cholesterol—(triglycerides/5), where “triglycerides/5” is used to represent very low-density lipoprotein-C (VLDL-C).

### Statistical Analysis

The obtained data were subjected to one-way analysis of variance using SPSS (version 22, USA). The replicate was considered as the experimental unit. Percentage data were transformed to arcsine to normalize data distribution. Differences within means of treatments (4 groups × 3 replicates) were tested by Tukey's test at *P* < 0.05. The polynomial contrast (linear or quadratic response) procedure was applied to evaluate the relationships that might exist among the treatment means when a significant effect was present. The statistical model for analysis data was Yij = μ + Ti + eij, where Yij= the observation, μ = the overall mean, Ti= the effect of treatment groups (i = 1, 2,. and 4), and eij= random error.

## Results

### Growth Performance and Feed Efficiency

The dietary effects of LZM on growth performance are presented in Table 2. The orthogonal contrast revealed that the effect of LZM was significant on the final body weight (FBW), body weight gain (BWG), feed intake (FI), and feed conversion ratio (FCR) regardless of the LZM dose (*P* < 0.05). FBW (*P* = 0.021) and BWG (*P* = 0.011) were linearly increased by increasing the LZM levels in the diets over the control group, and the values of 2256.1 to 2409.7 g for FBW and 1644 to 1788.2 g for BWG were recorded. Additionally, FI (*P* = 0.022) and FCR (*P* = 0.003) were quadratically decreased by increasing the LZM level over the control group.

**Table 2:** Effect of varying levels of chicken egg lysozyme (LZM) on the growth and feed efficiency of growing rabbits.

	**LZM0**	**LZM50**	**LZM100**	**LZM200**	**SEM^**1**^**	***P*-value^**5**^**	**Linear**	**Quadratic**
Initial BW (g)	612	613	611	621	0.353	NS	NS^4^	NS
Final BW (g)^2^^,^ ^3^	2,256^aA^	2,316^bB^	2343^bBC^	2,410^bC^	0.554	0.024	0.021	NS
BWG (g)	1,644^aA^	1,703^bB^	1,732^bBC^	1,788^bC^	0.426	0.001	0.011	NS
Total FI (g/period)	5,487^aC^	5,128^bB^	4,678^bA^	4,504^bA^	0.583	0.032	NS	0.022
Total FCR	3.34^aC^	3.01^bB^	2.70^bA^	2.50^bA^	0.042	0.025	NS	0.003

1 SEM, standard error of the mean.

2 Small superscript letters are significantly different within the same row depending on orthogonal contrasts.

3 Different capital letters are significantly different based on Tukey's test at (P < 0.05). BW, bodyweight (g); BWG, body weight gain (g); FI, feed intake (g, as feed); FCR, feed conversion ratio.

4 NS, not significant (P > 0.05).

5 *P-value based on the orthogonal contrast between the control treatment and sum of LZM treatments*.

One-way ANOVA showed that FBW and BWG were significantly higher by feeding LZM at 50, 100, and 200 mg per kg than rabbits fed the control diet and the group of rabbits that received 200 mg LZM/kg had the highest FBW and BWG. The group of rabbits fed 100 and 200 mg LZM/kg diet had lower total FI and FCR than the control and those fed 50 mg/kg and rabbits fed 50 mg/kg had lower FI and FCR than the control.

### Microbial Enumeration

Results of the cecum microbial counts for rabbits are presented in Table 3. The orthogonal contrast revealed that the effect of LZM was significant on cecum pH, the total bacterial count, total *Lactobacillus* bacteria, *E. coli* bacteria, and *Clostridium* spp regardless of the LZM dose (*P* < 0.05). The results indicated that the pH value was increased by increasing the LZM levels in a significant linear trend with values of 6.16 to 6.76 (*P* = 0.032). Concurrently, the total bacterial count (16.53–27.02 CFU/g) and the total *Lactobacillus* bacteria (5.08–11.42 CFU/g) were linearly increased by increasing the LZM levels (*P* = 0.021; *P* = 0.001). However, the total count of *E. coli* bacteria (1.61–0.91 CFU/g) and *Clostridium* spp (6.11–3.77 CFU/g) was decreased by increasing the level of LZM in a significantly linear trend (*P* = 0.001; *P* = 0.045). The lowest *E. coli* bacteria and *Clostridium* spp and the highest total bacterial count and the total *Lactobacillus* bacteria were observed in rabbits fed 200 mg LZM/kg diet (*P* < 0.05). The pH, total bacterial count, and total *Lactobacillus* bacteria were increased whereas *E. coli* bacteria and *Clostridium* spp were decreased by increasing the LZM levels.

**Table 3:** Effect of varying levels of chicken egg lysozyme (LZM) on cecum microbial activity of growing rabbits.

	**LZM0**	**LZM50**	**LZM100**	**LZM200**	**SEM^**1**^**	***P*-value^**6**^**	**Linear**	**Quadratic**
Cecum pH	6.16^aA^	6.60^bB^	6.72^bB^	6.79^bB^	0.051	0.001	0.032	NS^5^
*Clostridium* spp.^2^^,^ ^3^	6.11^aC^	4.32^bB^	4.14^bAB^	3.77^bA^	0.042	0.021	0.045	NS
*Escherichia coli* (×10^4^)^4^	1.61^aC^	1.05^bB^	1.02^bB^	0.91^bA^	0.013	0.003	0.001	NS
*Lactobacillus* (×10^5^)^4^	5.08^aA^	10.44^bB^	10.76^bB^	11.42^bC^	0.071	0.024	0.001	NS
Total bacterial count (×10^6^)^4^	16.5^aA^	22.0^bB^	24.7^bAB^	27.0^bC^	0.082	0.022	0.021	NS

1 SEM, standard error of the mean.

2 Small superscript letters are significantly different within the same row depending on orthogonal contrasts.

3 Different capital letters are significantly different based on Tukey's test at (P < 0.05).

4 Counts were presented in CFU/g cecal digesta.

5 NS, not significant (P > 0.05).

6 *P-value based on the orthogonal contrast between the control treatment and sum of LZM treatments*.

One-way ANOVA showed that rabbits fed LZM at 50, 100, and 200 mg/kg had significantly higher cecum pH than rabbits fed the control diet. Concurrently, the total bacterial count and the total *Lactobacillus* bacteria were higher in rabbits fed LZM at 50, 100, and 200 mg/kg than the control with the highest being in rabbits fed 200 mg/kg. Conversely, the total count of *E. coli* bacteria and *Clostridium* spp was lower in rabbits fed LZM at 50, 100, and 200 mg/kg than the control.

### Hematological and Blood Biochemical Parameters

The effects of dietary LZM on hematological variables are shown in Table 4. The orthogonal contrast revealed that the effect of LZM was significant on RBCs, Hb, PCV, WBCs, lymphocytes, heterophil, monocyte, and H/L ratio regardless of the LZM dose (*P* < 0.05). The simultaneous administration of LZM exerted a pronounced linear (*P* < 0.05) increase in blood parameters (RBCs, Hb, and PCV) by increasing the LZM levels in the diets. The highest values of RBCs, Hb, and PCV were recorded for rabbits fed the highest level (200 mg/kg diet) of LZM when compared to the control group (*P* = 0.032; *P* = 0.012; *P* = 0.003). Contrary to these results, insignificant changes in blood indices (MCV, MCH, and MCHC) were detected by LZM supplementation (*P* > 0.05). The data also demonstrated a significant linear increase in WBCs and lymphocytes(*P* = 0.002; *P* = 0.021), together with a significant linear decrease in heterophil, monocyte, and H/L ratio (*P* = 0.045; *P* = 0.031; *P* = 0.031). At the same time, eosinophil was non-significantly changed in LZM-treated groups compared to that of the control group (*P* > 0.05).

**Table 4:** Effect of varying levels of chicken egg lysozyme (LZM) on hematological variables of growing rabbits.

	**LZM0**	**LZM50**	**LZM100**	**LZM200**	**SEM^**1**^**	***P*-value^**5**^**	**Linear**	**Quadratic**
RBC (×10^6^/μl)^2^^,^ ^3^	4.20^aA^	4.51^bB^	4.51^bB^	4.66^bC^	0.042	0.002	0.032	NS^4^
Hb (g/dl)	8.53^aA^	9.06^bB^	9.40^bBC^	10.00^bC^	0.213	0.011	0.012	NS
PCV (%)	32.7^aA^	34.6^bB^	35.2^bB^	36.7^bC^	0.721	0.031	0.003	NS
MCV (fl)	55.8	58.9	57.2	57.0	1.242	NS	NS	NS
MCH (pg)	20.9	20.5	20.2	20.1	0.366	NS	NS	NS
MCHC (%)	36.0	35.9	35.6	35.5	0.314	NS	NS	NS
WBC (×10^3^/μl)	3.40^aA^	3.96^bB^	4.20^bC^	4.26^bC^	0.251	0.002	0.002	NS
Lymphocytes (%)	44.4^aA^	56.5^bB^	57.6^bB^	60.3^bC^	1.441	0.032	0.021	NS
Monocyte (%)	13.96^aC^	10.16^bB^	9.53^bA^	9.00^bA^	1.252	0.029	0.031	NS
Heterophil (%)	36.6^aC^	27.2^bB^	25.9^bA^	25.2^bA^	1.155	0.036	0.046	NS
Eosinophil (%)	2.26	1.80	1.76	1.66	0.221	NS	NS	NS
H/L ratio	0.82^aC^	0.48^bB^	0.45^bAB^	0.41^bA^	0.221	0.002	0.031	NS

1 SEM, standard error of the mean.

2 Small superscript letters are significantly different within the same row depending on orthogonal contrasts.

3 Different capital letters are significantly different based on Tukey's test at (P < 0.05).

4 NS, not significant (P > 0.05).

5 *P-value based on the orthogonal contrast between the control treatment and sum of LZM treatments*.

One-way ANOVA showed that rabbits fed LZM at 50, 100, and 200 mg/kg had significantly higher RBCs, WBCs, Hb, PCV, and lymphocytes than rabbits fed the control diet with the highest being in rabbits fed 200 mg/kg. However, heterophil, monocyte, and H/L ratio were higher in rabbits fed LZM at 50, 100, and 200 mg/kg than the control with the lowest being in rabbits fed 200 mg/kg.

The effects of LZM on serum biochemical analysis are shown in Table 5. The orthogonal contrast revealed that the effect of LZM was significant on serum total protein, globulin, A/G ratio, urea, creatinine, ALT, and AST regardless of the LZM dose (*P* < 0.05). The data explored a pronounced (*P* = 0.041) quadratic increase in serum total protein while globulin was linearly increased in rabbits fed LZM at varied levels (*P* = 0.012). However, the A/G ratio was linearly decreased (*P* = 0.002) in rabbits fed LZM at varied levels. Concerning liver function transaminase enzymes (ALT and AST), significantly linearly decreased levels in LZM-treated rabbit especially at the rates of 100 and 200 mg/kg diet (*P* = 0.041; *P* = 0.011) were revealed. Similarly, a significant linear decrease in urea and creatinine markers was observed in the LZM-treated rabbit groups. Regarding the albumin and lipid profile [total cholesterol, triglycerides, high-density lipoprotein (HDL), and low-density lipoprotein (LDL)], biomarker values in the current study did not reveal any significant differences in these parameters in LZM-supplemented rabbit groups comparable to the control group (*P* > 0.05).

**Table 5:** Effect of varying levels of chicken egg lysozyme (LZM) on biochemical blood variables of growing rabbits.

	**LZM0**	**LZM50**	**LZM100**	**LZM200**	**SEM^**1**^**	***P*-value^**5**^**	**Linear**	**Quadratic**
Total protein (g/dl)^2^^,^ ^3^	6.30^aA^	7.52^bB^	7.46^bB^	7.46^bB^	0.331	0.032	NS^4^	0.041
Albumin (g/dl)	4.05	3.89	3.70	3.42	0.131	NS	NS	NS
Globulin (g/dl)	2.25^aA^	3.63^bB^	3.76^bB^	4.04^bC^	0.442	0.001	0.012	NS
A/G ratio	1.80^aC^	1.07^bB^	0.98^bAB^	0.85^bA^	0.193	0.036	0.0021	NS
Creatinine (mg/dl)	1.18^aB^	0.88^bAB^	0.87^bA^	0.84^bA^	0.221	0.027	0.0012	NS
Urea (mg/dl)	4.40^aC^	3.66^bB^	3.53^bB^	3.30^bA^	1.442	0.002	0.0321	NS
AST (IU/L)	55.0^aC^	53.0^bB^	51.3^bAB^	50.7^bA^	1.113	0.043	0.0111	NS
ALT (IU/L)	47.0^aC^	36.6^bB^	34.6^bAB^	33.3^bA^	1.421	0.001	0.0411	NS
Cholesterol (mg/dl)	28.3	28.1	28.7	28.0	1.123	NS	NS	NS
Triglyceride (mg/dl)	74.7	78.7	73.3	73.3	1.0141	NS	NS	NS
v LDL (mg/dl)	16.9	16.7	16.7	16.7	0.212	NS	NS	NS
LDL (mg/dl)	2.03	1.66	2.34	2.14	0.331	NS	NS	NS
HDL (mg/dl)	11.3	10.7	11.7	11.2	0.611	NS	NS	NS

1 SEM, standard error of the mean.

2 Small superscript letters are significantly different within the same row depending on orthogonal contrasts.

3 Different capital letters are significantly different based on Tukey's test at (P < 0.05).

4 NS, not significant (P > 0.05).

5 *P-value based on the orthogonal contrast between the control treatment and sum of LZM treatments*.

One-way ANOVA showed that rabbits fed LZM at 50, 100, and 200 mg/kg had significantly higher total protein and globulin than rabbits fed the control diet. However, A/G ratio, creatinine, urea, ALT, and AST were lower in rabbits fed LZM at 50, 100, and 200 mg/kg than the control with the lowest being in rabbits fed 200 mg/kg.

## Discussion

The use of LZM in the diets of animals is one of the best ways to gain weight and raise the ability of animals to cope with diseases (19). Dietary LZM can be considered as a feasible feed additive due to its functions as an immunostimulant and antibacterial potential, and it plays a vital role in the host–pathogen relationship change and can be suggested for further research for the treatment of COVID-19 (9). As a result of LZM supplementation in the rabbit diet in the present study, the body weight and body weight gain increased with improved feed efficiency. The orthogonal contrast revealed that LZM significantly increased the final body weight (FBW) and body weight gain (BWG) but decreased the feed intake (FI) and feed conversion ratio (FCR) regardless of the LZM dose. The obtained results are in agreement with those reported by (20, 30). In the same line, Oliver and Wells (30) stated that including LZM to weaning pigs' diets improved feed conversion. The positive effect of LZM on BW, BWG, FI, and FCR in the current study may be due to the improved gut antioxidant and antimicrobial activities together with enhanced digestibility of nutrients in rabbits gut, which can be attributed to increased absorption of nutrients in the intestine (31, 32). Also, the improved growth performance may be attributed to enhanced immune responses by the beneficial microbiota. Brundige et al. (33) reported that 18 known serum metabolites were increased by dietary LZM. Four of these metabolites are methionine, threonine, and hydroxyproline, indicating increased protein synthesis and skeletal growth and, in turn, increased growth performance by dietary LZM. The improvement in the FCR and growth performance in the current study can be explained by the richness of LZM with amino acid (33), in addition to its antioxidant and antimicrobial properties, which could increase microbial activity and feed efficiency in the gut of rabbits.

The rabbit's cecal microbial population plays an essential role in the digestion process and improve both health and digestive efficiency, as its population represents 40% of the gastrointestinal tract volume (34, 35). The cecal pH value in rabbits depends on some factors like diet amount and composition. The changes in pH value could reflect the changes in organic acid accumulated in the ingesta. The orthogonal contrast revealed that the effect of LZM significantly increased the cecum pH, the total bacterial count, and total *Lactobacillus* bacteria but decreased *E. coli* bacteria and *Clostridium* spp regardless of the LZM dose. *Lactobacillus* is a beneficial bacterium for animals found in the cecum, which helps effectively in digestion and local immunity (36). The improvement in *Lactobacillus* count by feeding LZM may be due to the antimicrobial, antioxidant, anti-inflammatory, and immune-modulatory properties of LZM (16), which was used as an indicator for good healthy states of rabbits. Dietary LZM decreased *E. coli* and *Clostridium* spp., which can be attributed to the antibacterial properties that led to inhibition of the growth of pathogenic bacteria, with beneficial effects on the host health (20, 31). Our results are in line with the findings of Gong (37) and Brundige et al. (33), who mentioned that used LZM decreased the number of *E. coli* in young pigs. Furthermore, the mode of action of LZM as an antibacterial agent is hydrolyzation of the peptidoglycan in the pathogenic bacterial cell wall (23).

The detection of the hematological and biochemical indices provides essential diagnostic tools about the health conditions of domestic animals fed varied feed additives (8, 38). Chicken egg LZM is an interesting antibiotic peptide and growth enhancer with a remarkable bacteriolytic ability that can be used efficiently in rabbit feeding (16, 37). In the current study, dietary LZM exerted a pronounced improvement in the total number of RBC, Hb, and PCV regardless of the LZM dose. This improvement in RBCs count could be due to the general enhancement of the health status and physiological well-being of the rabbits fed LZM (38). The increased WBCs and lymphocytes (%) may be attributed to the ability of LZM to cause some degree of improvement in immunity. The orthogonal contrast revealed a significant increase in serum total protein and globulin levels regardless of the LZM dose. This finding was supported by elevated serum total protein and globulin concentrations and low H/L ratio, indicating a better immune status. Moreover, the best H/L ratio was recorded for rabbits fed LZM compared to the control group. In agreement with our results, Fritz et al. (39) mentioned that LZM supplementation enhanced the immunity of chickens because of the antioxidant and immune status improvements. In the same line, Zou et al. (19) reported that LZM might have immune-modulating properties by modulating the production of antibodies (i.e., IgA) in the serum and intestinal mucosa of growing pigs. Similar results were confirmed by Oliver et al. (32), who documented an enhanced immune response by supplementing LZM to pigs' diets.

No adverse effects for dietary LZM on liver and kidney function variables were detected in treated rabbits. The safety of LZM supplementation is evidenced by the non-significant elevation of serum ALT and AST activities, which indicates the improved overall health and liver-protective effects of LZM. The orthogonal contrast revealed that the urea, creatinine, ALT, and AST values were significantly decreased regardless of the LZM dose. The results also suggested that LZM is a good source of antioxidant that can protect cells from free radicals and may reduce the toxicity that can protect liver health (40). Similarly, a non-significant increase in urea and creatinine as kidney injury markers was recorded in rabbit fed with LZM compared to the control group. These results are in the same line with Oliver et al. (32), who found that LZM consumption increased the growth rate and decreased the circulating urea, in addition to an increase in protein accretion.

Regarding the lipid profile (TC, TGs, HDL, and LDL) biomarkers in the current study, the findings did not reveal any significant differences in these biomarkers in rabbit fed LZM comparable to the control group. The obtained results indicate no direct correlation between LZM feeding and lipid profiles. However, we evaluated the lipid profile because they are synthesized in the liver and are considered as indicators for liver performance. These results further confirm the role of LZM as a functional feed additive with nutritive and health-improving properties (41).

## Conclusions

Dietary LZM in rabbits' diets leads to improved growth rate and feed efficiency and reduces potential pathogen shedding. The total count of *Escherichia coli* and *Clostridium* count were decreased, while the total bacterial count and the total count of *Lactobacilli* had increased considerably by dietary LZM. Chicken egg LZM could also benefit growing rabbits with better immune status without adverse side effects on hematological parameters. Hence, LZM is required at the rate of 100–200 mg/kg diet as a potential feed additive and a friendly alternative for antibiotics in rabbit feed.

## Data Availability Statement

The raw data supporting the conclusions of this article will be made available by the authors, without undue reservation.

## Ethics Statement

The animal study was reviewed and approved by Faculty of Agriculture, Kafrelsheikh University, Egypt.

## Author Contributions

All authors listed have made a substantial, direct and intellectual contribution to the work, and approved it for publication.

## Conflict of Interest

The authors declare that the research was conducted in the absence of any commercial or financial relationships that could be construed as a potential conflict of interest.
